# Association between Antibiotic Exposure and the Risk of Rash in Children with Infectious Mononucleosis: a Multicenter, Retrospective Cohort Study

**DOI:** 10.1128/aac.00249-23

**Published:** 2023-05-23

**Authors:** Rui Zhang, Zhen Mao, Chang Xu, Wen Wang, Joey Sum-wing Kwong, Minjie Xu, Yi Song, Tianyi Lv, Zhiyuan Teng, Ruifeng Zhong, Hui Liu, Yang Liu, Qin Wang, Ying Wang, Yuan Zhang, Shuya Chen, Xiuli Chai, Rui He, Wenyi Zheng, Jiaxing Zhang

**Affiliations:** a Department of Pharmacy, Guizhou Provincial People’s Hospital, Guiyang, China; b Department of Pharmacy, Guiyang Maternal and Child Health Care Hospital, Guiyang, China; c College of Public Health, Anhui Medical University, Hefei, China; d Chinese Evidence-Based Medicine Center and Cochrane China Center, West China Hospital, Sichuan University, Chengdu, China; e Global Health Nursing, Graduate School of Nursing Science, St. Luke’s International University, Tokyo, Japan; f Department of Pharmacy, Liupanshui Maternal and Child Health Care Hospital, Liupanshui, China; g Department of Pharmacy, Tongren City People’s Hospital, Tongren, China; h Department of Pharmacy, Xingyi People’s Hospital, Xingyi, China; i Department of Pharmacy, Guiyang Second People’s Hospital, Guiyang, China; j Department of Pharmacy, Jinsha People’s Hospital, Bijie, China; k Department of Pharmacy, Anshun People’s Hospital of Guizhou Province, Anshun, China; l Department of Pharmacy, The Second Affiliated Hospital of Guizhou Medical University, Kaili, China; m Department of Pharmacy, People’s Hospital of Qianxinan Prefecture, Xingyi, China; n Department of Pharmacy, GuiHang GuiYang Hospital, Guiyang, China; o Department of Pharmacy, The First People’s Hospital of Bijie, Bijie, China; p Department of Pharmacy, The First People’s Hospital of Guiyang, Guiyang, China; q Department of Pharmacy, Qiannan Buyei and Miao Autonomous Prefecture People’s Hospital, Duyun, China; r Department of Laboratory Medicine, Experimental Cancer Medicine, Karolinska Institute, Stockholm, Sweden

**Keywords:** infectious mononucleosis, antibiotics, aminopenicillins, amoxicillin, rash, cohort study

## Abstract

Present evidence suggests that the administration of antibiotics, particularly aminopenicillins, may increase the risk of rash in children with infectious mononucleosis (IM). This retrospective, multicenter cohort study of children with IM was conducted to explore the association between antibiotic exposure in IM children and the risk of rash. A robust error generalized linear regression was performed to address the potential cluster effect, as well as confounding factors such as age and sex. A total of 767 children (aged from 0 to 18 years) with IM from 14 hospitals in Guizhou Province were included in the final analysis. The regression analysis implied that exposure to antibiotics was associated with a significantly increased incidence of overall rash in IM children (adjusted odds ratio [AOR], 1.47; 95% confidence interval [CI], ~1.04 to 2.08; *P *= 0.029). Of 92 overall rash cases, 43 were probably related to antibiotic exposure: two cases (4.08%) in the amoxicillin-treated group and 41 (8.15%) in the group treated with other antibiotics. Regression analysis indicated that the risk of rash induced by amoxicillin in IM children was similar to that induced by other penicillins (AOR, 1.12; 95% CI, ~0.13 to 9.67), cephalosporins (AOR, 2.45; 95% CI, ~0.43 to 14.02), or macrolides (AOR, 0.91; 95% CI, ~0.15 to 5.43). Antibiotic exposure may be associated with an increased risk of overall rash in IM children, but amoxicillin was not found to be associated with any increased risk of rash during IM compared to other antibiotics. We suggest that clinicians be vigilant against the occurrence of rash in IM children receiving antibiotic therapy, rather than indiscriminately avoiding prescribing amoxicillin.

## INTRODUCTION

Infectious mononucleosis (IM), a viral disease primarily caused by Epstein-Barr virus (EBV), usually occurs in children, adolescents, and young adults (1). It is characterized by the typical triad fever, pharyngeal inflammation, and cervical lymphadenopathy (2). Since fever, pharyngitis, fatigue, and lymphadenopathy are common presenting symptoms in the outpatient setting, IM is often mistaken for other entities, such as acute bacterial tonsillitis (3). However, despite the fact that IM is a benign self-limiting disease that does not require antimicrobial therapy, previous studies have demonstrated that 54.30% to 84.00% of IM patients continue taking antibiotics empirically throughout the course of their disease (4–12).

Rashes have been reported in IM patients following antibiotic use, particularly with aminopenicillins (13). In earlier studies conducted between 1967 and 1972 (4–8), ~42.22% to 100% of IM patients treated with ampicillin developed a rash. As a result, IM has been considered a contraindication for ampicillin or amoxicillin. Both the *Nelson Textbook of Pediatrics* (14) and *Principles and Practice of Infectious Disease* (15) stated that the incidence of rash caused by ampicillin or amoxicillin in IM was up to 80%. However, later studies from 2013 to 2018 (9–11) reported the prevalence of rash in IM patients receiving amoxicillin as only ~12.50% to 29.51%, suggesting that its true incidence may be much lower than that for ampicillin. Moreover, in addition to aminopenicillins, a similar rash was observed in IM patients treated with other antibiotics, including other penicillins (6–8, 16), tetracyclines (4–6), sulfonamides (8), cephalosporins (11), clindamycin (11), cephalexin (17), macrolides (18), levofloxacin (19), and piperacillin-tazobactam (20). Interestingly, rashes can also occur with IM in the absence of antibiotic exposure, and the general incidence is ~4.20% to 13.00% (3, 14, 21). Considering that eruptions are usually erythematous, macular, and popular or morbilliform and involve the trunk and extremities as occasional symptoms of IM, it may be difficult for physicians to distinguish a disease-associated rash from a drug allergy (22, 23). A review (24) of 17 case reports suggested the necessity of reassessing the long-held belief that antibiotic-induced skin rash occurs with high incidence in IM patients. Recently, three cohort studies (9–11) demonstrated that no significant difference in the incidence of antibiotic-related rash was found between antibiotic-treated and -untreated IM patients, which calls into question the higher rate of rash in IM patients receiving antibiotics. Moreover, a systematic review (25) including 10 cohort studies and 1,325 IM patients also concluded that the administration of amoxicillin in IM patients might not increase the risk of rash (risk ratio [RR], 0.85; 95% confidence interval [CI], ~0.53 to 1.33; *P *= 0.45) and the incidence of rash in IM patients with aminopenicillin treatment might not be higher than that with other antibiotic treatments. However, this conclusion remains to be further verified by more studies with larger sample sizes and better methodological designs, considering the small sample sizes, the limited representativeness, and poor comparability of cohorts of the included studies.

In the context of limited evidence about this question, this study aimed to explore the association between antibiotic exposure in IM children and the risk of rash and in particular, to compare the effect of amoxicillin and other antibiotics.

## RESULTS

### Patient baseline characteristics.

**FIG 1:**
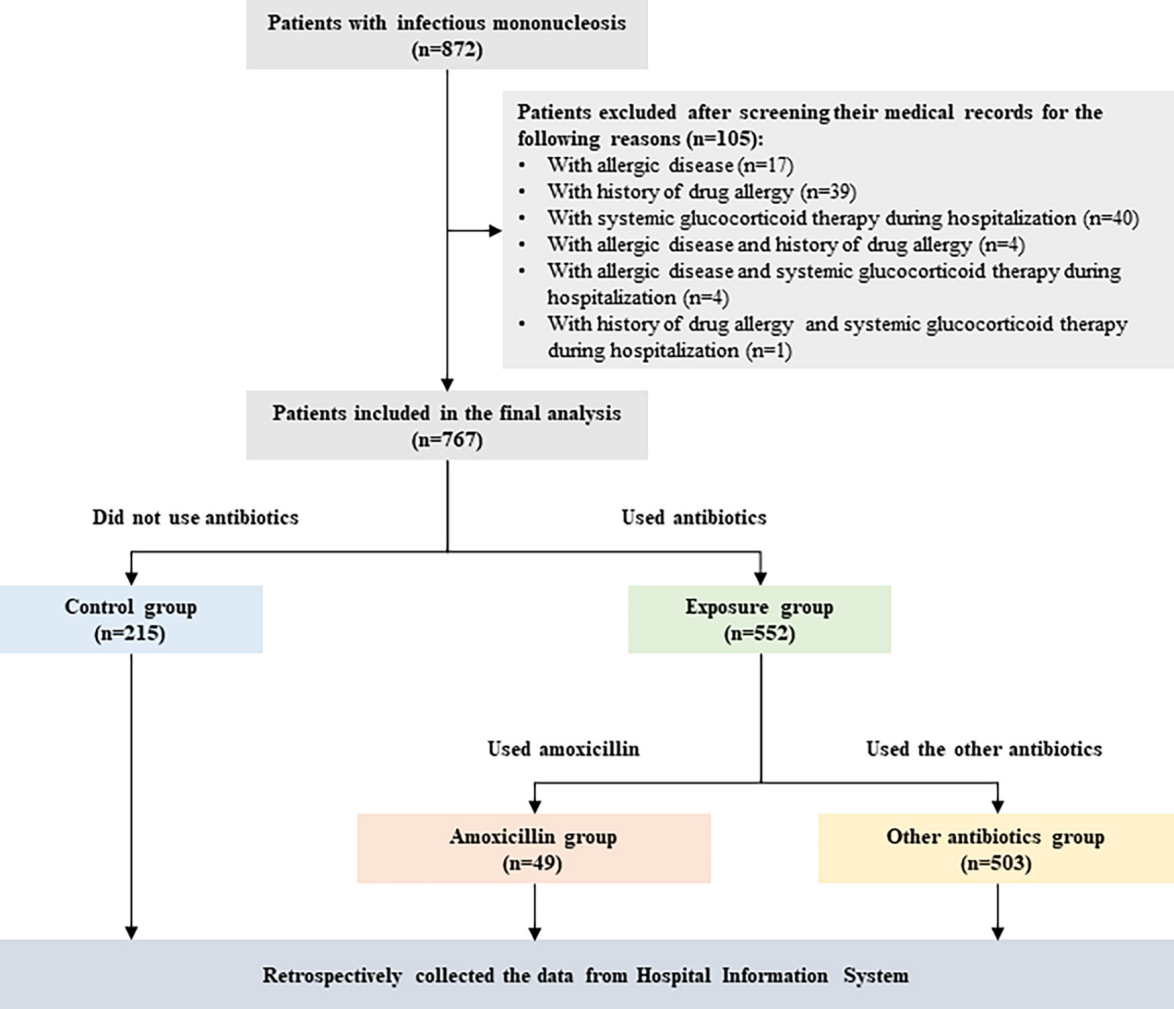
Study design schematic.

During the study period, a total of 872 children from 14 hospitals were diagnosed with IM. After exclusion, 767 children with hospital duration of ~7 to 14 days were included in the analysis (Fig. 1). There was a significant difference in age between the excluded and included patients (*P *= 0.005; seer Table S6 in the supplemental material). As presented in Table 1, the children were mainly aged from 0 to 6 years old (79.53%), and 60.63% were boys. We identified 552 patients who were treated with antibiotics during their disease course, among whom 49 were treated with amoxicillin, and 503 received other antibiotics. The remaining 215 participants without antibiotic treatment were regarded as controls. Table 1 shows that no significant differences existed in the patient age and sex regardless of the use and the type of antibiotics.

**TABLE 1:** Characteristics of the patients

Characteristic	No. of patients (%)			*P*	No. of patients (%)		*P*
	Total	With no antibiotics	With antibiotics		With amoxicillin	With other antibiotics	
No. of patients	767	215	552		49	503	
Sex				0.914			0.305
Female	302 (39.37)	84 (39.07)	218 (39.49)		16 (32.65)	202 (40.16)	
Male	465 (60.63)	131 (60.93)	334 (60.51)		33 (67.35)	301 (59.84)	
Age (yrs)				0.592^*a*^			0.746^*a*^
0–6	610 (79.53)	175 (81.40)	435 (78.80)		40 (81.63)	395 (78.53)	
7–12	146 (19.04)	36 (16.74)	110 (19.93)		8 (16.33)	102 (20.28)	
13–18	11 (1.43)	4 (1.86)	7 (1.27)		1 (2.04)	6 (1.19)	
Overall rash				0.154		
No	675 (88.01)	195 (90.70)	480 (86.96)			
Yes	92 (11.99)	20 (9.30)	72 (13.04)			
Antibiotic-associated rash							0.412^*a*^
No					47 (95.92)	462 (91.85)	
Yes					2 (4.08)	41 (8.15)	

a Fisher’s exact test.

### Overall rash.

**FIG 2:**
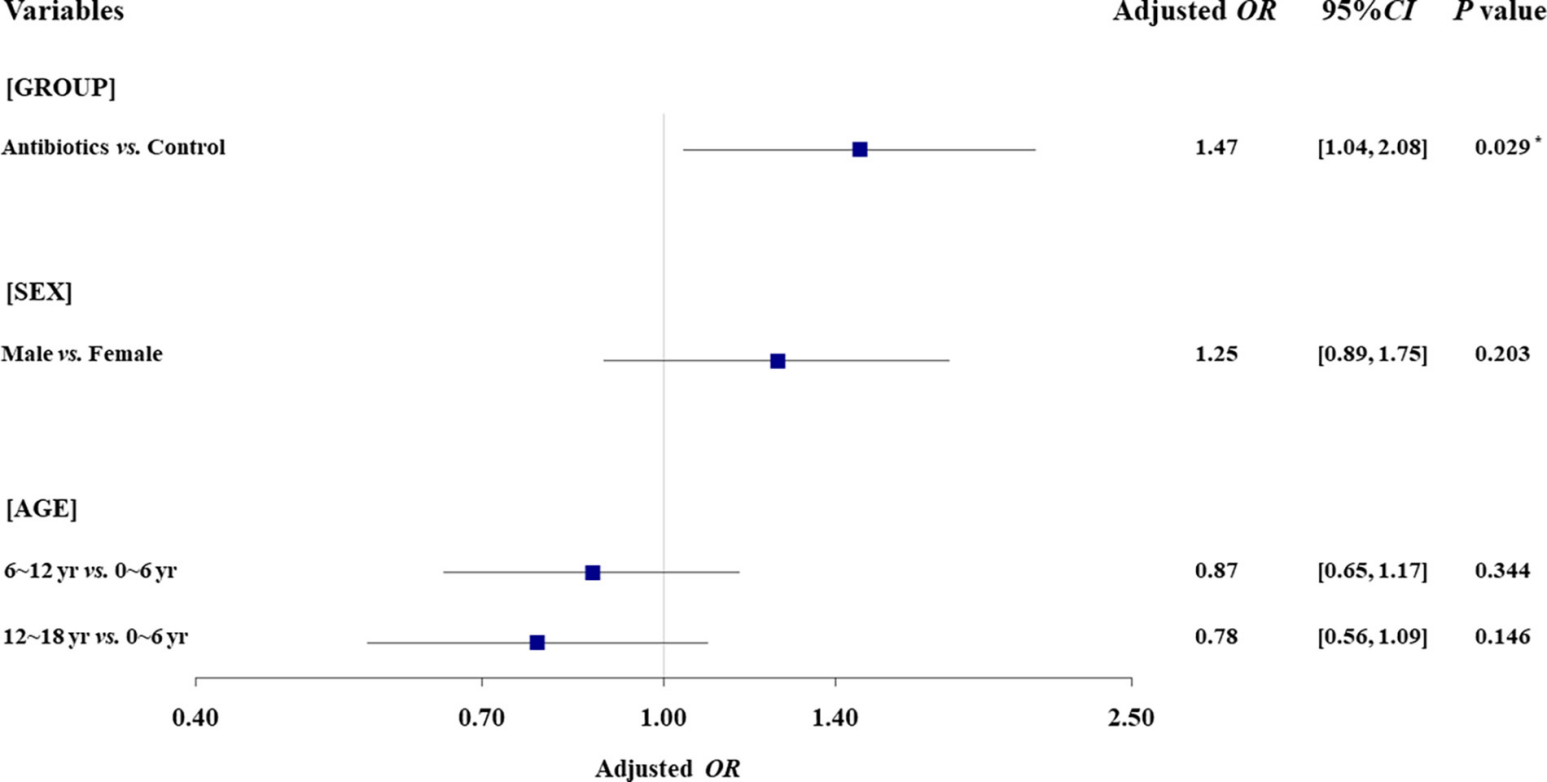
Results of multivariate analyses of overall rash. *, indicates *P* < 0.05.

As shown in Table 1, 72 (13.04%) of the treated patients developed a rash, compared to 20 (9.30%) of the untreated patients (OR, 1.46; 95% CI, ~0.87 to 2.47; Table 2). Nevertheless, a generalized linear model (Table 2 and Fig. 2) implied that exposure to antibiotics was associated with a significantly increased incidence of overall rash in IM children after controlling for both age and sex (adjusted OR [AOR], 1.47; 95% CI, ~1.04 to 2.08; Table 2).

**TABLE 2:** Results of univariate and multivariate (generalized linear model) analyses^*a*^

Outcome	Exposure vs comparator	Unadjusted			Adjusted^*b*^		
		OR	95% CI	*P*	OR	95% CI	*P*
Overall rash	Antibiotics vs control	1.46	0.87–2.47	0.154	1.47	1.04–2.08	0.029^*c*^
Antibiotic-associated rash	Amoxicillin vs other antibiotics	0.48	0.05–1.95	0.412	0.48	0.10–2.31	0.360

a OR, odds ratio; CI, confidence interval.

b Adjusted for age, sex, and hospital.

c *P *< 0.05.

### Antibiotic-associated rash.

**FIG 3:**
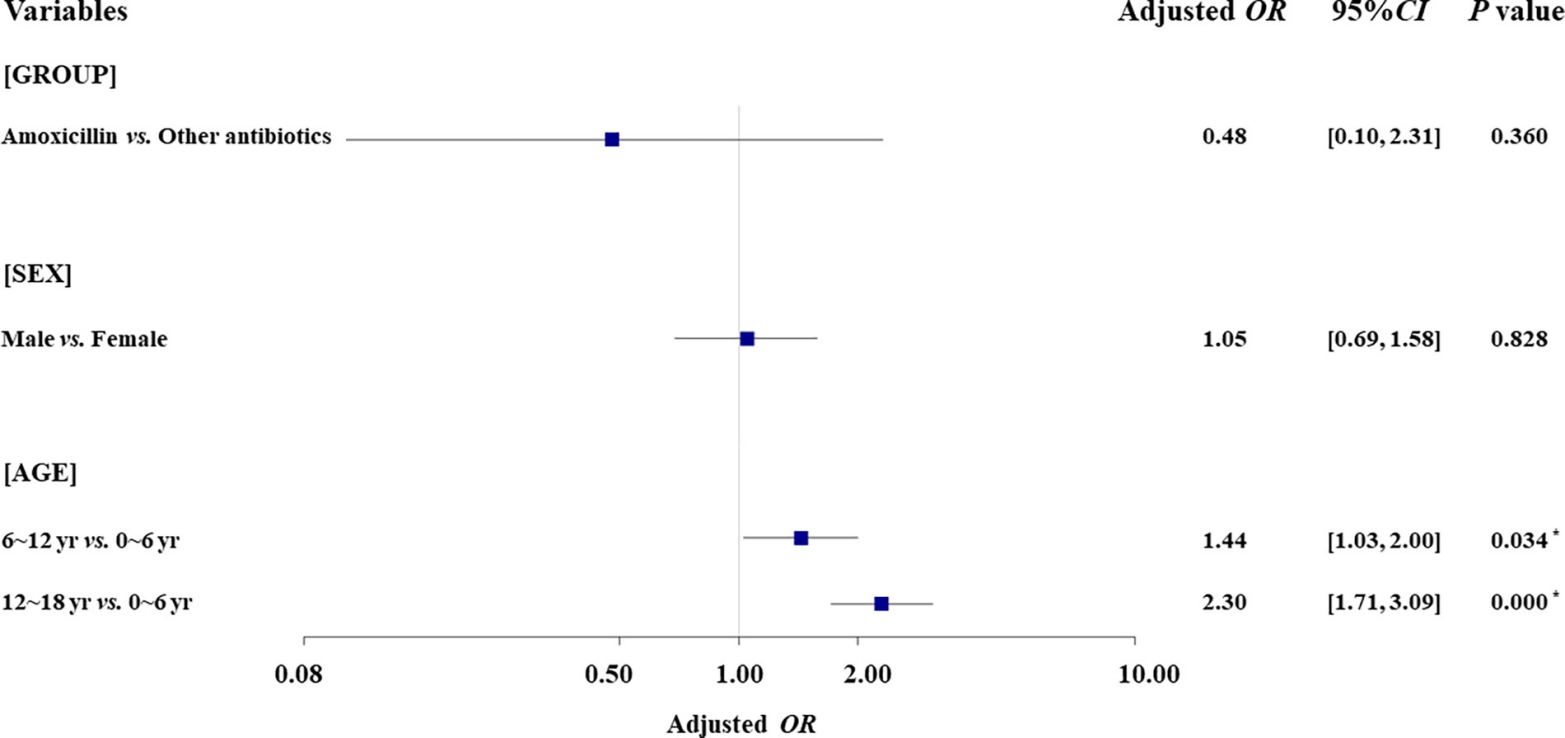
Results of multivariate analyses of rashes associated with amoxicillin versus other antibiotics. *, indicates *P* < 0.05.

It should be noted that only 43 of 92 overall rash cases were graded with scores of 5 to 7 by pharmacists and probably related to the antibiotic exposure: 2 cases (4.08%) in the amoxicillin-treated group and 41 (8.15%) in the group treated with other antibiotics. Both unadjusted and adjusted analyses (Table 2) indicated that the risk of antibiotic-associated rash was not significantly higher in IM patients treated with amoxicillin than in those treated with the other antibiotics (OR, 0.48; 95% CI, ~0.05 to 1.95; AOR, 0.48; 95% CI, ~0.10 to 2.31). However, patient age would be one independent factor associated with the antibiotic-associated rash. Compared to children aged 0 to 6 years old, IM patients aged 6 to 12 and 12 to 18 years old seemed to be at higher risk for antibiotic-associated rash (AOR, 1.44; 95% CI, ~1.03 to 2.00; and AOR, 2.30; 95% CI, ~1.71 to 3.09, respectively; Fig. 3).

A total of 433 children were given only one type of antibiotic during their hospitalization. Table 3 shows that 1, 2, 20, and 2 children (3.23%, 3.85%, 7.12%, and 2.90%) who took amoxicillin, other penicillins, cephalosporins, and macrolides developed an antibiotic-associated rash, respectively. Multivariate analysis (Fig. S1) indicated that the risk of rash induced by amoxicillin in IM children was similar to that induced by other penicillins, cephalosporins, or macrolides (AOR, 1.12; 95% CI, ~0.13 to 9.67; AOR, 2.45; 95% CI, ~0.43 to 14.02; and AOR, 0.91; 95% CI, ~0.15 to 5.43, respectively) after adjusting for age and sex.

**TABLE 3:** Incidence of different antibiotic-associated rash (*n* = 433)

Antibiotic(s)	No. of patients		Incidence (%)
	Treated	With antibiotic-associated rash	
Amoxicillin/amoxicillin-clavulanate	31	1	3.23
Other penicillins	52	2	3.85
Cephalosporins	281	20	7.12
Macrolides	69	2	2.90

### Sensitivity analysis.

Considering that antibiotic-associated rash was a rare event in this study, we performed a sensitivity analysis. As presented in Table S7, the results of the Firth logistic regression model were consistent with those of the generalized linear model.

## DISCUSSION

The results of this retrospective, multicenter cohort study show that administration of antibiotics can be associated with an increased risk of overall rash in IM children, but amoxicillin was not found to be associated with any increased risk of antibiotic-induced rash during IM compared to other antibiotics (other penicillins, cephalosporins, or macrolides). Furthermore, older age may be an independent risk factor for IM children to develop antibiotic-associated rash.

The incidence of overall rash among IM children treated with antibiotics in this study was 13.04%, which was lower than that recorded in most studies (4–6, 8, 9, 12) but close to that reported by Davis (14.89%) (7), Hocqueloux et al. (15.32%) (10), and Misirlioglu et al. (16.67%) (11). Furthermore, only 46.70% of rashes in this study overall were probably associated with antibiotic exposure, so the actual incidence (7.79%) of antibiotic-induced rash was much lower. One possible reason for the lower incidence of rash in our study may be the different race of the patients; this study also excluded patients with comorbid allergic disease and history of drug allergy, which would increase the risk of rash. However, the underlying mechanism of the rash is still not well understood, although several relevant studies have been performed (16, 26–29). The most commonly proposed mechanism is a transient virus-mediated immune alteration, resulting in the development of a reversible, delayed-type hypersensitivity reaction to the antibiotics (24).

Notably, the inconsistent conclusions in the current evidence introduce doubt about the ability of antibiotics to increase the risk of rash in IM patients (3–11). A systematic review (25) indicated that the incidence of rash in IM patients treated with and without antibiotics were not significantly different on the basis of recently published studies (RR, 1.02; 95% CI, ~0.75 to 1.37), which is not in agreement with the results of the current multicenter cohort study (AOR, 1.47; 95% CI, ~1.04 to 2.08). This inconsistency may be attributable to the different study population, outcome definition, or analysis method. Specifically, we only included Chinese children with IM and without allergic disease or history of drug allergy; the rashes in our study were antibiotic induced, disease associated, or related to other factors; and we performed a multivariate analysis model to adjust for demographic confounders (age and sex). In contrast, as the systematic review mentioned, most prior studies (4–8) were conducted before 1976 with small sample sizes and without detailed methods, while a few studies conducted after 2010 were assessed using the Newcastle-Ottawa scale as having a high risk of bias due to their methodological limitations; therefore, the reliability of their conclusions is compromised (24). Altogether, with its larger sample size and precise statistical methods, the present multicenter cohort study provides more powerful evidence about the research question.

As to the antibiotic-induced rash, this study found that the risk of rash related to amoxicillin or other antibiotics was similar in IM patients (AOR, 0.48; 95% CI, ~0.10 to 2.31), in line with the results of a previous systematic review and meta-analysis (25). Furthermore, 18 children (36.73%) treated with amoxicillin in this study were concomitantly treated with cephalosporins or macrolides, which would increase the risk of rash in the amoxicillin group but strengthen the robustness of the above conclusion. According to further analyses among children receiving only one type of antibiotic, the risk of amoxicillin-induced rash was comparable to that of other penicillins, cephalosporins, or macrolides, which is consistent with the results of Misirlioglu et al. (11). Several studies (4–6, 9, 10) reported that ampicillin (aminopenicillin) exposure is related to the significantly elevated absolute risk of rash, and another review (30) agreed that instead of ampicillin, amoxicillin should be reconsidered without fear of it causing a rash, especially in cases of penicillin V intolerance. However, we could not verify these findings because no patients were treated with ampicillin in our study.

Our study has several strengths. First, unlike previous studies, which were case reports (13) and small uncontrolled studies (24), the present study was a large cohort study in Chinese children with IM to explore the risk of rash induced by treatment with antibiotics. Second, all prior studies with a control (4–12) performed only univariate analysis without ensuring the comparability of cohorts; in contrast, we utilized the general linear model to control for potential confounding factors such as age, which was found to be an independent risk factor for antibiotic-associated rash in IM children. Third, we found that the excluded population was different from the included population, which further reinforced our decision to exclude them as a different population who may have introduced bias.

Nevertheless, this study was an observational study based on retrospective data, which should be interpreted in this light. No randomization method was applied in our observational study in view of ethical concerns; hence, selection bias existed in the study. A multivariate statistical method was used to control for confounding factors; however, it is likely that some unknown or undetermined residual confounding factors were present. Although we planned a comparison between aminopenicillin and other antibiotics, the data regarding amoxicillin and ampicillin were not adequate. Due to the restrictions of drug instruction, only a small number of children with IM were treated with amoxicillin, and no clinicians prescribed ampicillin. As a result, we were unable to explore whether aminopenicillin would increase the risk of rash compared to other antibiotics. Furthermore, due to inaccurate and incomplete data, misclassification bias is common in retrospective studies. In this study, the accuracy of outcome measurement was highly dependent on the level of detail in patient progress notes recorded in the electronic medical record (EMR) system; for instance, few physicians recorded details about the rash such as location, morphology, and characteristics, so the likelihood of bias in the records cannot be ignored when interpreting the results. The strength of inference about the causality was thus weakened, given the nature of a retrospective observational study.

As we know, there is a clinical overlap in the presenting features of IM and acute bacterial tonsillitis, which is commonly caused by group A beta-hemolytic Streptococcus. Therefore, many clinicians prescribe antibiotics only based on symptoms before the underlying cause of the illness has been well identified. Nevertheless, due to the advice of the National Institute for Health and Care Excellence (NICE) against the use of amoxicillin in IM patients (31), the pediatricians in our study always selected other penicillins, cephalosporins, or macrolides as an alternative. In fact, present evidence has revealed that these antibiotics do not have a lower risk of rash than amoxicillin, challenging the long-held viewpoint of clinicians. As amoxicillin is a common option in empirical treatment of pediatric infectious diseases, when treating IM children with antibiotics, we suggest that clinicians be vigilant against the occurrence of rash in IM children receiving antibiotic therapy, rather than indiscriminately avoiding prescribing amoxicillin.

## MATERIALS AND METHODS

We followed the STROBE (*st*rengthening the *r*eporting of *ob*servational studies in *e*pidemiology) statement to conduct and report the present cohort study (see Table S1 in the supplemental material) (32).

### Study design and setting.

This retrospective, multicenter cohort study was performed in 14 hospitals across Guizhou Province, China (see the list of hospitals in Table S2). These hospitals cover 31.97 million people over 38.5 million people and thus is expected to represent the capital tertiary care public hospitals in the province. The health information system (HIS) at these hospitals was initially developed in 2015, and a stable electronic medical record (EMR) system was established in these hospitals which integrated the HIS with the laboratory information system and stored individual patient health care and medical information. The EMR system contains information stored in structured or semistructured formats (i.e., patient demographics, prescriptions, and diagnoses) and unstructured formats (e.g., history of present illness and progress notes). All data were collected from the EMR system of these hospitals between 1 January 2020 and 31 December 2020 in accordance with predesigned case report forms (CRFs). This study was approved by the Ethics Committee of the Guizhou Provincial People’s Hospital (2017066; Table S3) and conducted in accordance with the Declaration of Helsinki (33).

### Study population.

Children (aged 0 to 18 years old) hospitalized with IM were consecutively included from 1 January 2020 and 31 December 2020. We excluded patients who met any of the following criteria: comorbid allergic disease; history of drug allergy; received systemic glucocorticoid therapy during hospitalization. The main reason was that these factors are associated with the risk of rash—excluding them benefits confounding control. It should be noted that such exclusion may have reduced the representativeness of the sample and further biased the results; to test whether the exclusion impact the results, the baseline characteristics of the excluded against the included patients were compared (see Results). As a retrospective cohort study without any planned intervention, no additional informed consent was obtained from the patients after approval from the institutional ethical committee, according to the confidentiality protocol.

### Exposure and controls.

Exposure was identified by prescription review, in which a prescription for antibiotics was considered drug exposure, while the control was defined as no antibiotics use. Information regarding drug use (drug name, dose, usage, administration time, and duration) was obtained from the prescription data, and children were considered to be in the exposure group if they had received at least one dose of antibiotics during their hospitalization. Patients with exposure were further divided into two subgroups based on the prescribed antibiotics (i.e., amoxicillin or other antibiotics).

### Outcomes.

The primary outcome was the incidence of rash, which includes overall and antibiotic-associated rash. Overall rash, identified in the light of patients’ medical records from the EMR system, refers to the rash all patients developed during their illness both before and after antibiotic exposure. Antibiotic-associated rash was defined as a rash that occurred following the administration of antibiotics that could not be explained by other reasons, such as the patient’s clinical condition, other treatment, or concomitant medication. Two pharmacists independently assessed whether there was a causal relationship between the occurrence of rash and the antibiotic exposure according to the adverse drug reaction (ADR) probability scale (Table S4) developed by Naranjo (34). The ADR was assigned to a probability category from the total score as follows: definite, ≥9; probable, 5 to 8; possible, 1 to 4; doubtful, ≤0 (34). After a group discussion among the research team members, a rash with a total score of more than 5 was considered an antibiotic-associated rash. Any disagreement was resolved by discussion until consensus was achieved.

### Covariates.

Based on clinical evidence and biological rationale, we defined demographic characteristics (age and sex) as potentially important confounders, which were extracted from personal hospitalization records for all subjects. Diagnosis information (infectious mononucleosis) was identified using the International Classification of Disease (ICD) 10 code. Concomitant medications that could cause rash were identified from the prescription data.

### Data collection and management.

The investigators manually reviewed all the information of every included patient one by one. The data were recorded and crosschecked by two independent investigators who had undergone a unified online training about the details of the study protocol and CRFs. To avoid detection bias, the investigators who assessed the overall rash did not know the previous prescriptions of patients. Missing data would occur when the physicians omitted the demographic information of patients in the medical record. In that case, the investigators would contact the patients or their parents to obtain the exact information.

### Sample size estimation.

We estimated the minimum sample size (*n*) needed for the incidence of rash using the formula below (35). This was done to ensure that sufficient power would be achieved when calculating the incidence of rash. We set five plausible values for the incidence (*P*) (0.01, 0.05, 0.1, 0.3, and 0.5) to estimate the required sample size using the 95% CI of the incidence of rash that adhered to previously reported data and a margin of error (*E*) of 0.1 and 0.05. These assumptions generated the required sample size, varying from 4 to 384, with the incidence of 0.5 generating the largest sample size of 384 (Table S5):

$$n=\frac{1.96^{2}P(1-P)}{E^{2}}$$

### Data analysis.

Stata 14.0 was utilized to perform statistical analyses, and the significance level was set as 0.05 (36). We summarized the baseline characteristics and the incidence of rash using descriptive statistics. Categorical variables were presented as numbers with percentages. Chi-square tests (or Fisher’s exact tests) or Mann-Whitney U tests were used for group comparisons (37). To compare the risk of rash between two groups, a generalized linear model was performed by treating the overall rash as the dependent variable and the exposure factor and other important covariates (age and sex) as independent variables. Results were presented as the odds ratio (OR) with 95% CI, considering that the OR is a “portable” effect (38). The robust-error estimator was utilized in the presence of data aggregation at the study center (hospital) level (39). Considering that in some strata, the events might be rare, a sensitivity analysis using penalized maximum likelihood logistic regression was employed to address potential “spare data bias” (40). The same statistical analysis methods were also applied to explore the effect of the antibiotic type on an antibiotic-associated rash.
