# Supplementary material for: Association between Antibiotic Exposure and the Risk of Rash in Children with Infectious Mononucleosis: a Multicenter, Retrospective Cohort Study
Source: Antimicrob Agents Chemother. 2023 May 23;67(6):e00249-23. doi: 10.1128/aac.00249-23 (PMC10269065; doi:10.1128/aac.00249-23)
Supplement: Supplemental file 1 — Supplemental material. Download aac.00249-23-s0001.docx, DOCX file, 5.5 MB [file aac.00249-23-s0001.docx]

**Table S1 STROBE Statement—Checklist of items that should be included in reports of *cohort studies***

|  | Item No | Recommendation | Reporting Location |
| --- | --- | --- | --- |
| **Title and abstract** | 1 | (*a*) Indicate the study’s design with a commonly used term in the title or the abstract | Abstract |
|  |  | (*b*) Provide in the abstract an informative and balanced summary of what was done and what was found | Abstract |
| Introduction | | |  |
| Background/rationale | 2 | Explain the scientific background and rationale for the investigation being reported | Introduction |
| Objectives | 3 | State specific objectives, including any prespecified hypotheses | Introduction |
| Methods | | |  |
| Study design | 4 | Present key elements of study design early in the paper | Methods |
| Setting | 5 | Describe the setting, locations, and relevant dates, including periods of recruitment, exposure, follow-up, and data collection | Methods |
| Participants | 6 | (*a*) Give the eligibility criteria, and the sources and methods of selection of participants. Describe methods of follow-up | Methods |
|  |  | (*b*) For matched studies, give matching criteria and number of exposed and unexposed | N/A |
| Variables | 7 | Clearly define all outcomes, exposures, predictors, potential confounders, and effect modifiers. Give diagnostic criteria, if applicable | Methods |
| Data sources/ measurement | 8* | For each variable of interest, give sources of data and details of methods of assessment (measurement). Describe comparability of assessment methods if there is more than one group | Methods and Table S3 |
| Bias | 9 | Describe any efforts to address potential sources of bias | Methods  Discussion |
| Study size | 10 | Explain how the study size was arrived at | Methods  Table S4 |
| Quantitative variables | 11 | Explain how quantitative variables were handled in the analyses. If applicable, describe which groupings were chosen and why | N/A |
| Statistical methods | 12 | (*a*) Describe all statistical methods, including those used to control for confounding | Methods |
|  |  | (*b*) Describe any methods used to examine subgroups and interactions | Methods |
|  |  | (*c*) Explain how missing data were addressed | Methods |
|  |  | (*d*) If applicable, explain how loss to follow-up was addressed | N/A |
|  |  | (*e*) Describe any sensitivity analyses | Methods |
| Results | | |  |
| Participants | 13* | (a) Report numbers of individuals at each stage of study—eg numbers potentially eligible, examined for eligibility, confirmed eligible, included in the study, completing follow-up, and analysed | Methods  Results  Figure 1  Table S5 |
|  |  | (b) Give reasons for non-participation at each stage | Figure 1 |
|  |  | (c) Consider use of a flow diagram | Figure 1 |
| Descriptive data | 14* | (a) Give characteristics of study participants (eg demographic, clinical, social) and information on exposures and potential confounders | Results  Table 1 |
|  |  | (b) Indicate number of participants with missing data for each variable of interest | N/A |
|  |  | (c) Summarise follow-up time (eg, average and total amount) | N/A |
| Outcome data | 15* | Report numbers of outcome events or summary measures over time | Results |
| Main results | 16 | (*a*) Give unadjusted estimates and, if applicable, confounder-adjusted estimates and their precision (eg, 95% confidence interval). Make clear which confounders were adjusted for and why they were included | Results  Table 2 |
|  |  | (*b*) Report category boundaries when continuous variables were categorized | Results  Table 1  Figure 2 |
|  |  | (*c*) If relevant, consider translating estimates of relative risk into absolute risk for a meaningful time period | N/A |
| Other analyses | 17 | Report other analyses done—eg analyses of subgroups and interactions, and sensitivity analyses | Results  Figure S1  Table S7 |
| Discussion | | |  |
| Key results | 18 | Summarise key results with reference to study objectives | Discussion |
| Limitations | 19 | Discuss limitations of the study, taking into account sources of potential bias or imprecision. Discuss both direction and magnitude of any potential bias | Methods  Discussion |
| Interpretation | 20 | Give a cautious overall interpretation of results considering objectives, limitations, multiplicity of analyses, results from similar studies, and other relevant evidence | Discussion |
| Generalisability | 21 | Discuss the generalisability (external validity) of the study results | Methods  Discussion |
| Other information | | |  |
| Funding | 22 | Give the source of funding and the role of the funders for the present study and, if applicable, for the original study on which the present article is based | Abstract |

*Give information separately for exposed and unexposed groups.

**Note:** An Explanation and Elaboration article discusses each checklist item and gives methodological background and published examples of transparent reporting. The STROBE checklist is best used in conjunction with this article (freely available on the Web sites of PLoS Medicine at http://www.plosmedicine.org/, Annals of Internal Medicine at http://www.annals.org/, and Epidemiology at http://www.epidem.com/). Information on the STROBE Initiative is available at http://www.strobe-statement.org.

**Table S2 The list of hospitals participating this study**

| **No.** | **Name of hospital** | **Region** | **Number of patients** |
| --- | --- | --- | --- |
| 1 | Guizhou Provincial People’s Hospital  (Principal Investigator and Lead Institution) | Guiyang | 386 |
| 2 | Guiyang Maternal and Child Health Care Hospital | Guiyang | 132 |
| 3 | Liupanshui maternal and Child Health Care Hospital | Liupanshui | 46 |
| 4 | Tongren City People's Hospital | Tongren | 40 |
| 5 | Xingyi People's Hospital | Qianxinan | 31 |
| 6 | Guiyang Second People's Hospital | Guiyang | 25 |
| 7 | Jinsha People’s Hospital | Bijie | 24 |
| 8 | Anshun People’s Hospital of Guizhou Province | Anshun | 14 |
| 9 | The Second Affiliated Hospital of Guizhou Medical University | Qiandongnan | 14 |
| 10 | People’s Hospital of Qianxinan Prefecture | Qianxinan | 13 |
| 11 | GuiHang GuiYang Hospital | Guiyang | 12 |
| 12 | The First People’s Hospital of Bijie | Bijie | 11 |
| 13 | The First People's Hospital of Guiyang | Guiyang | 10 |
| 14 | Qiannan Buyei and Miao Autonomous Prefecture People's Hospital | Qiannan | 9 |

**Table S3. Ethical Approval Documentation (Chinese)**


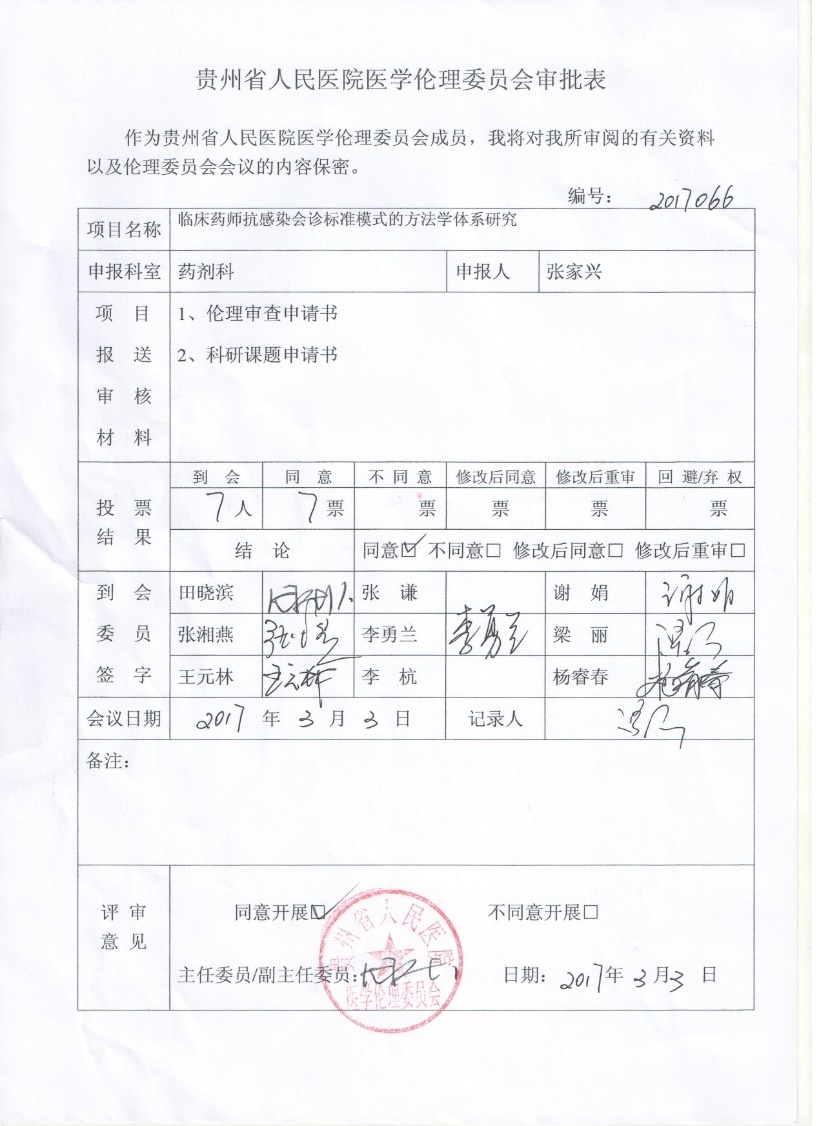


**Table S3. Ethical Approval Documentation (Translation to English)**

**Approval form of the Medical Ethics Committee of Guizhou Provincial People’s Hospital**

As a member of the Medical Ethics Committee of Guizhou Provincial People’s Hospital, I will keep secret for the materials which I reviewed and the content of the conference.

The Number: 2017066

| Title of the project | Methodological research on the standard procedure of clinical pharmacists’ consultation for infectious diseases. | | | | | |
| --- | --- | --- | --- | --- | --- | --- |
| Department | Department of Pharmacy | | | Applicant | Zhang Jiaxing | |
| The materials for ethical review | 1. Application for ethical review  2. Application for the study projects | | | | | |
| The results of voting | Attendance  (n) | Agree  (n) | Disagree  (n) | Agree after the project was revised  (n) | Review after the project was revised  (n) | Avoidance / abstention  (n) |
|  | 7 | 7 |  |  |  |  |
|  | Conclusion | | Agree√ Disagree□  Agree after the project was revised□  Review after the project was revised□ | | | |
| Member of the ethics committee | Tian Xiaobing |  | Zhang Qian |  | Xie juan |  |
|  | Zhang Xiangyan |  | Li Yonglan |  | Liang Li |  |
|  | Wang Yuanlin |  | Li Hang |  | Yang Ruichun |  |
| Conference date | 3/3/2017 | | | Conference recorder | Wang Han | |
| Remark: None | | | | | | |
| Review comments | Agree to conduct this project√  Disagree to conduct this project□  Chairman: Date: 3/3/2017 | | | | | |

**Table S4. ADR probability scale**

|  | Yes | No | Do not know | Score |
| --- | --- | --- | --- | --- |
| 1. Are there previous conclusive reports on this reaction? | +1 | 0 | 0 |  |
| 2. Did the adverse event appear after the suspected drug was administered? | +2 | -1 | 0 |  |
| 3. Did the adverse reaction improve when the drug was discontinued or specific antagonist was administered? | +1 | 0 | 0 |  |
| 4. Did the adverse reaction reappear when the drug was readministered? | +2 | -1 | 0 |  |
| 5. Are there alternative causes (other than the drug) that could on their own have caused the reaction? | -1 | +2 | 0 |  |
| 6. Did the reaction reappear when a placebo was given? | -1 | +1 | 0 |  |
| 7. Was the drug detected in the blood (or other fluids) in concentrations known to be toxic? | +1 | 0 | 0 |  |
| 8. Was the reaction more severe when the dose was increased, or less severe when the dose was decreased? | +1 | 0 | 0 |  |
| 9. Did the patient have a similar reaction to the same or similar drugs in any previous exposure? | +1 | 0 | 0 |  |
| 10. Was the adverse event confirmed by any objective evidence? | +1 | 0 | 0 |  |

The ADR was assigned to a probability category from the total score as follows: definite ≥ 9, probable 5 to 8, possible 1 to 4, doubtful ≤ 0.

**Table S5. Sample size estimation**

| **Error(E)** | **Incidence (P)** | | | | |
| --- | --- | --- | --- | --- | --- |
|  | **0.01** | **0.05** | **0.1** | **0.3** | **0.5** |
| **0.05** | 16 | 73 | 139 | 323 | 384 |
| **0.1** | 4 | 19 | 35 | 81 | 96 |

**Table S6. The characteristics of patients included and excluded. (n = 872)**

|  | **Included patients**  **(n=767)** | **Excluded patients**  **(n=105)** | ***P*** |
| --- | --- | --- | --- |
| **Sex** |  |  | 0.530 |
| **female**  **male** | 302 (39.37%)  465 (60.63%) | 38 (36.19%)  67 (63.81%) |  |
| **Age, years** |  |  | 0.005 ^a, *^ |
| **0-6**  **7-12**  **13-18** | 610 (79.53%)  146 (19.04%)  11 (1.43%) | 90 (85.71%)  10 (9.52%)  5 (4.77%) |  |

**^a^: Fisher’s exact test; *: *P*<0.05.**

**Table S7. The results of sensitivity analyses by Firth-logistic regression model**

| **Outcome** | **Exposure vs. Comparator** | ***Unadjusted*** | | | ***Adjusted ^a^*** | | |
| --- | --- | --- | --- | --- | --- | --- | --- |
|  |  | ***OR*** | **95%*CI*** | ***P*** | ***OR*** | **95%*CI*** | ***P*** |
| **Antibiotic-associated rash** | *Amoxicillin vs. Other antibiotics* | 0.59 | 0.16~2.18 | 0.425 | 0.59 | 0.16~2.18 | 0.429 |
|  | *Other penicillin vs. Amoxicillin* | 1.01 | 0.13~8.00 | 0.995 | 0.94 | 0.12~7.52 | 0.955 |
|  | *Cephalosporins vs. Amoxicillin* | 1.59 | 0.29~8.72 | 0.591 | 1.69 | 0.31~9.28 | 0.544 |
|  | *Macrolides vs. Amoxicillin* | 0.75 | 0.10~5.96 | 0.788 | 0.76 | 0.09~6.16 | 0.800 |

***OR*: *Odds ratio*; *CI*: *Confidence interval*; ^#^: adjusted for age and sex.**


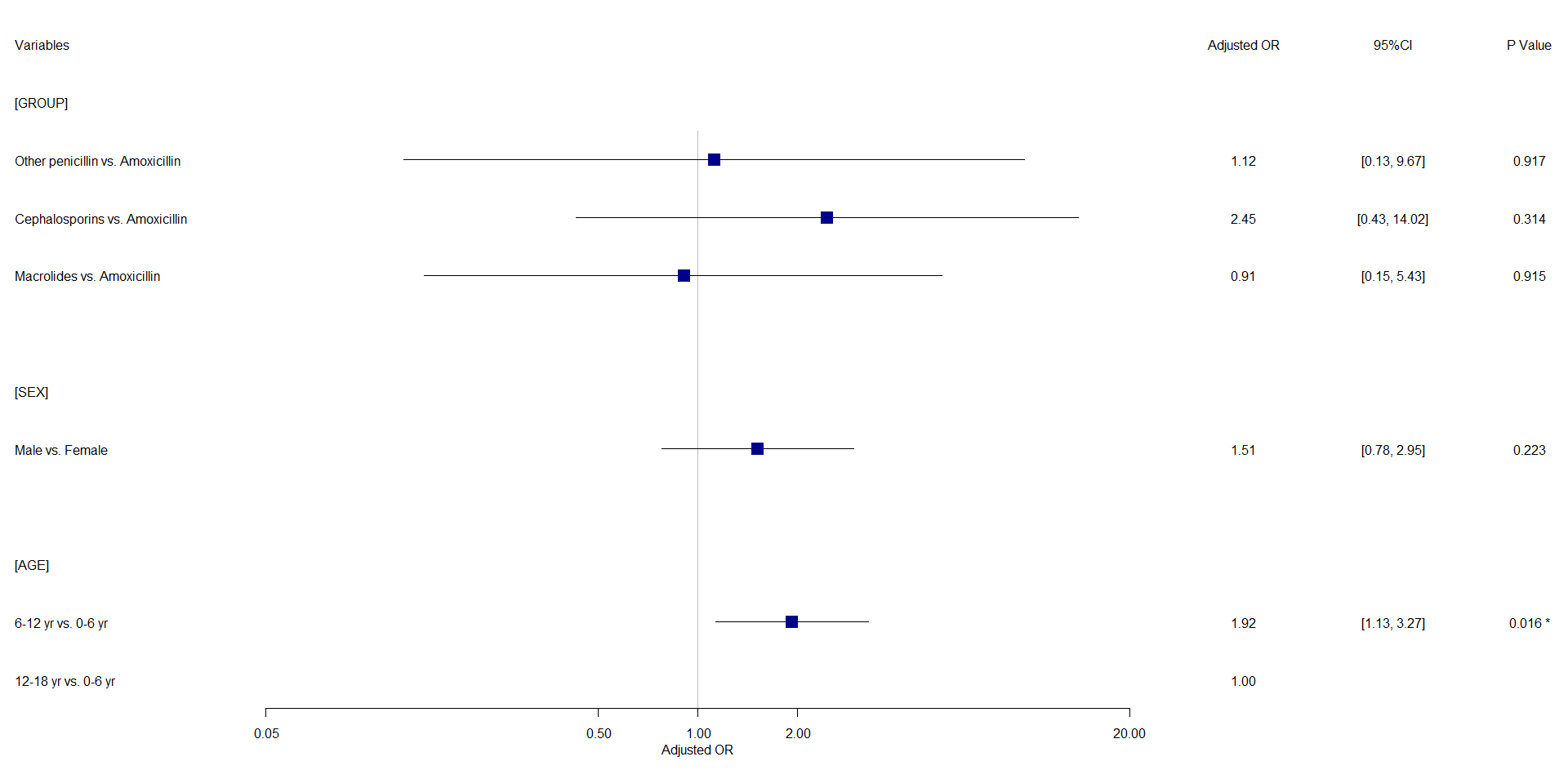


**Figure S1. The results of multivariate analyses of rash induced by different types of antibiotics.**
